# Successful Third Kidney Transplantation in a Nigerian Kidney Transplant Center: A Case Report

**DOI:** 10.7759/cureus.32521

**Published:** 2022-12-14

**Authors:** Olalekan O Olatise, Stephen O Asaolu, Michael O Muoka, Adegboyega E Faponle, Martin C Igbokwe, Ikechukwu Anosike, Uzodimma E Onwuasoanya, Adaku T Olatise, Kumar R Vasanth

**Affiliations:** 1 Department of Medicine/Nephrology Unit, Zenith Medical and Kidney Centre, Abuja, NGA; 2 Department of Clinical Research, Zenith Medical and Kidney Centre, Abuja, NGA; 3 Department of Surgery, Urology Unit, Zenith Medical and Kidney Centre, Abuja, NGA; 4 Department of Medicine, Zenith Medical and Kidney Centre, Abuja, NGA

**Keywords:** covid-19 vaccine, desensitization, third, challenges, kidney transplantation

## Abstract

Patients with end-stage renal disease have limited options in the course of their management. Kidney transplantation (KT) remains the gold standard for the management of renal replacement therapy. There is increasing evidence supporting the viability of third and fourth KTs. Due to the complexities of carrying out a successful third KT and the scarcity of living organ donors, it is not a common procedure in Nigeria's few renal transplant centres. To date, there is no reported case of a successful third KT in Nigeria. Here, we present the first reported case of a third KT carried out in Nigeria on a 44-year-old hypertensive, hepatitis B-infected, non-diabetic male patient. He had the first living donor KT eight years ago, which he lost due to poor immunosuppressive medication adherence. He then had a second living donor, KT, four years ago. Both KTs were from altruistic donors and were performed in the same hospital outside Nigeria. He developed allograft nephropathy after receiving the AstraZeneca COVID-19 vaccine 16 months ago and lost the second graft as a result. He was worked up and transplanted at our centre. Third kidney transplantation can be performed successfully despite the challenges of human leukocyte antigen (HLA) sensitization, antibiotic resistance, and surgical placement of the graft.

## Introduction

Kidney transplantation (KT) remains the gold standard for the management of renal replacement therapy [1]. It gives patients with end-stage kidney disease (ESKD) a better quality of life compared with haemodialysis and is also more cost-effective in the long term while affording the patient increased survival [2]. Despite massive improvements in short-term graft survival following a KT in recent decades, long-term survival rates have not improved significantly. More patients are returning to dialysis and therefore being relisted for KT following renal allograft failure [3]. Graft failure in a living recipient following KT could be due to risk factors for ESKD in the native kidneys, immunological factors (antibody-mediated rejections (ABMR), T-cell-mediated rejections, microvascular inflammation without antibodies), and non-immunological factors (donor age, donor cardiovascular morbidity, delayed graft function, infections, calcineurin inhibitor [CNI] toxicity) [4].

There is increasing evidence supporting the viability of third and fourth KTs. Although long-term patients and graft outcomes are inferior when compared with first KTs, the short-term outcomes are comparable [1,5,6]. Even in HLA-incompatible living donors, the short-term and long-term outcomes are similar [7].

Since the first third KT in 1978 [8], thousands of third KTs have been carried out in centres in developed countries. Due to the complexities of carrying out a successful third KT and the scarcity of living organ donors, it is not a common procedure in Nigeria's few renal transplant centres. To date, there has been no reported case of a successful third KT in Nigeria. Here, we present the first reported case of a third KT carried out in Nigeria.

## Case presentation

A 44-year-old hypertensive, hepatitis B-infected, non-diabetic male patient, came to our hospital for a third kidney transplant. He was diagnosed with ESKD (secondary to hypertensive nephropathy) nine years ago when he presented to another hospital with complaints of persistent generalized body weakness. Prior to his ESKD diagnosis, he had been hypertensive for 16 years and claimed to be adherent to his antihypertensive medications. He had the first living donor KT eight years ago, which he lost due to poor immunosuppressive medication adherence. He then had a second living donor, KT, four years ago. Both KTs were performed at the same hospital in India.

The current allograft nephropathy started after he received the AstraZeneca COVID-19 vaccine 16 months ago. A kidney biopsy was done, and it revealed a pattern consistent with focal segmental glomerulosclerosis (FSGS). His renal function deteriorated rapidly over four months, and he resumed a thrice-weekly dialysis session. He was on dialysis for 11 months before coming to our centre for KT.

We commenced his work-up for KT in our centre. During his serology workup, he was found to be infected with hepatitis B, and his viral load was 78.6 copies/µl. He was placed on entecavir (0.5 mg daily) for six months with constant monitoring, after which his hepatitis B viral load became undetectable. His first cousin, who was of the same blood group as him, offered to donate a kidney to him. Crossmatch results between him and his donor were negative for both B and T lymphocytes. He had a 3/6 HLA mismatch with high antibody titre levels. He has donor-specific antibody class I B18:01 levels of 9,948 MFIs, class II DR15:03 levels of 14,282 MFIs, and class II DP10 levels of 17,279 MFIs. He had a total of five plasma exchanges with 5 g of intravenous (IV) immunoglobulin for each exchange session and also had IV rabbit anti-thymocyte globulin (ATG) for desensitization. His donor-specific antibody levels were less than 1000 MFIs before transplantation. Crossmatching carried out between him and his donor was negative for both B and T lymphocytes.

On the day of the surgery, both the donor and the recipient were taken to the twin operating rooms of the hospital. The renal allograft was surgically removed from the donor through a mini-flank incision, as previously described [9]. During the donor workup, computed tomography angiography revealed a solitary renal artery and renal vein on the kidneys on both sides. The excised kidney was immediately immersed in ice and perfused with 500 mL to 1 L of cold perfusion fluid (0-4 °C) until the effluent from the renal vein was clear and devoid of blood. The perfusion fluid used was the University of Wisconsin solution. The first warm ischaemic time was 74 seconds, while the cold ischaemic time was 57 minutes, 2 seconds.

**Figure 1:**
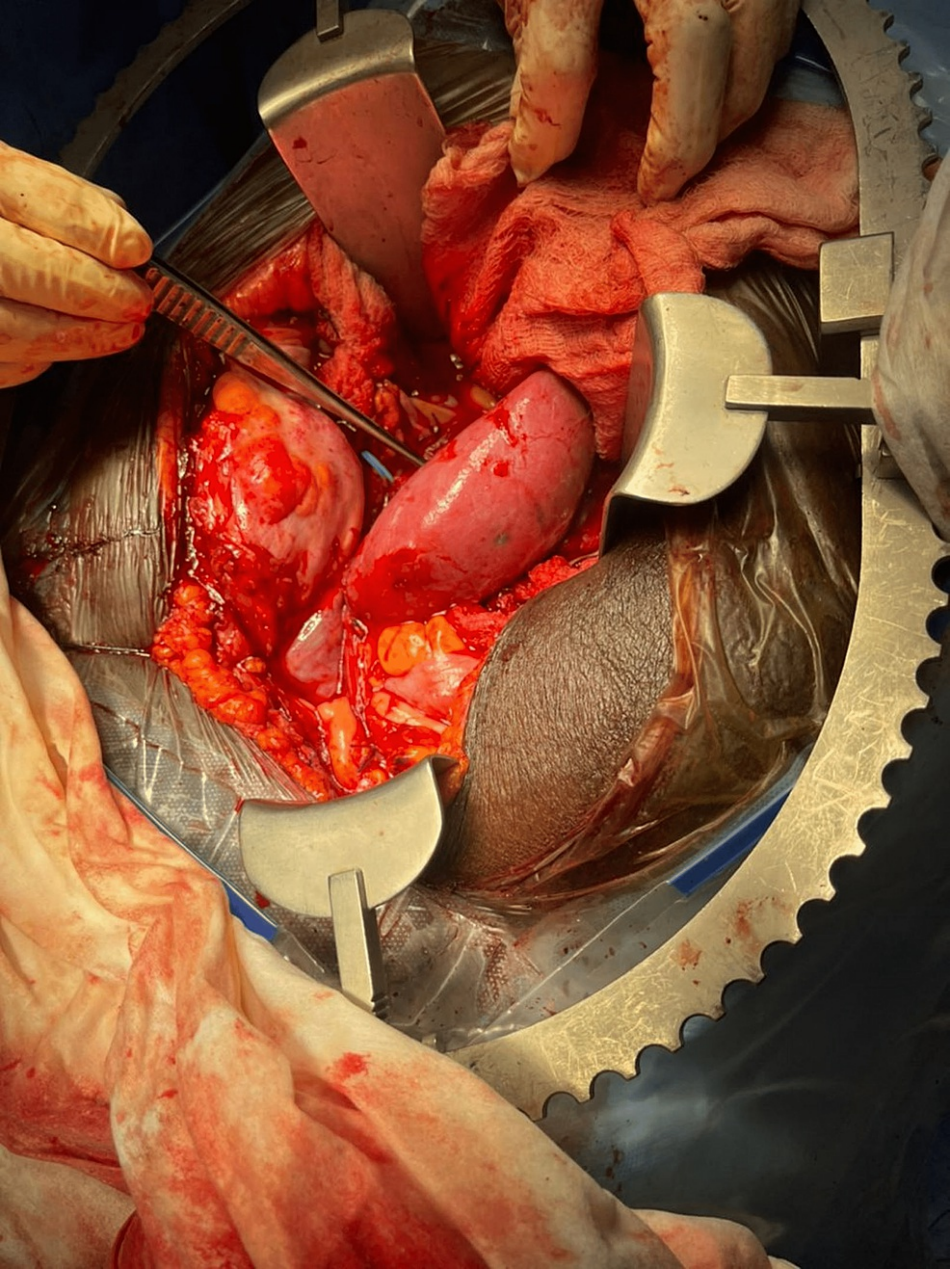
Image showing the new renal allograft.

The recipient was placed in a supine position, and spinal epidural anaesthesia was administered. The approach to the recipient vessels for anastomosis with the donor vessels was through the previous right Gibson incision, which was developed into the peritoneal cavity superior and medial to the previous first transplanted kidney, which was left in situ. The common iliac artery and vein were mobilized and prepared. The donor renal artery was anastomosed to the right common iliac artery, while the donor renal vein was anastomosed to the right common iliac vein using prolene 6/0 sutures. The clamps were released, and the anastomoses were checked. The allograft was turgid, pink, and making good urine, showing good perfusion to the kidney (Figure 1). The second warm ischaemic time was 52 minutes, 11 seconds. The ureter was anastomosed to the anterior wall of the urinary bladder and tunnelled to discourage vesico-ureteric reflux with a double J stent placed in situ. Adequate haemostasis was secured. The wound was closed in layers over the peritoneal drain, with the last layer closed using staples. He suspected high spinal pressure leading to cardiac arrest while under combined spinal and epidural anaesthesia during the surgery. He was promptly resuscitated using cardiac massage, a shot of epinephrine (1 mg), and a shot of ephedrine (15 mg). After recovery, he was intubated, and general anaesthesia was administered for the rest of the surgery.

He was transferred to the high dependency unit (HDU) immediately after the surgery; his PCV post-surgery was 21%, and he was transfused with two units of blood. He stayed in the HDU for seven days before being transferred to the ward. His post-transplant immunosuppressive regimen was MMF, TAC, Prednisolone, tab Valgancyclovir 450 mg daily, tab Cotrimoxazole 480 mg daily, oral Nystatin 100,000 iu, tab Isoniazid 300 mg daily, and tab Pyridoxine 25 mg daily. The trough level tacrolimus target in the first 2-3 weeks was 10-12ng/ml and the target in 3-4 months was 8-10ng/ml. Post-operatively, we continued to monitor his hepatitis B viral load.

**Table 1:** The urine output, serum creatinine, serum urea levels, and packed cell volume (PCV) in the first 21 days post-transplant. POD: post operative day, PCV: packed cell volume.

Day	Urine output (mls)	Creatinine (µmol/L)	Urea (mmol/L)	PCV (%)
Before transplant	Nil	1361	31.6	25.0
Day of surgery	780	1167	29.1	21.0
POD 1	13,160	873	17.5	25.3
POD 2	14,980	511	18.3	23.4
POD 3	6,535	305	14.0	23.0
POD 7	2,655	253	17.9	20.9
POD 14	2,300	371	12.1	22.5
POD 21	3,750	181	8.7	27.2

His wound drain was removed (POD 34), and he was discharged home the next day for a two-day clinic follow-up.

## Discussion

Despite the increasing advances in medical and surgical sciences and practise in west and central Africa, the area of transplantation has not received a commensurate expansion. Only a few centres possess the skills and infrastructure to offer kidney transplantation. This is in part, due to the complexities of commencing and maintaining an active kidney transplantation programme. It is therefore important to report that we successfully performed a third transplant in our centre.

The aetiology of the allograft failure in this patient was acute humoral and cellular rejection following administration of the adenovirus-vectored SARS-CoV-2 vaccine (AstraZeneca vaccine). Similar findings have been reported in several cases [10-12]. Organ rejection following COVID-19 vaccine administration in transplant patients is a possible but extremely rare phenomenon [13]. Transplant recipients are among the high-risk group of patients who were recommended to receive the COVID-19 vaccine. Recipients of kidney transplantation are at increased risk for infections (including COVID-19), and even when vaccinated, their chances for seroconversion may be reduced owing to their immunosuppressed state [14]. However, the risk-benefit ratio of vaccination outweighs the risk of having COVID-19.

In a review of 2,492 third kidney transplants carried out in the United States between 1995 and 2009, the graft outcome in the patients was slightly inferior when compared with the first kidney transplant [1], although a third kidney transplant still offers the patients a significantly higher patient survival advantage when compared with being on the waitlist. The duration of the second graft in these patients was a determinant of the survival of the third graft [1].

Several challenges are associated with performing the third transplant on a patient, which can be medical or surgical. A large proportion of repeat transplant patients develop anti-HLA antibodies, leading to a positive crossmatch result. These HLA-sensitized patients require several rounds of desensitization before they can be transplanted, and failure to do this can lead to significant fibrosis and scarring in the short term [15,16] and acute or chronic antibody-mediated rejection in the medium term [17,18]. Desensitization protocols vary from centre to centre and depend on the anti-HLA antibodies present in the patient; they often include a combination of plasmapheresis, IVIg, and rituximab. Sensitized patients benefit from desensitization and transplantation as this reduces their risk of mortality by half when compared with those who remain on dialysis [19]. After successful kidney transplantation is carried out in a sensitized patient, the physician must continually watch out for ABMR, as desensitization does not completely eradicate the risk for ABMR in highly sensitized patients [18].

Our patient was transplanted into the peritoneal cavity, close to the site of the first kidney transplant. Usually, the first and second kidney transplantations are performed on each side of the ipsilateral iliac fossa of the recipient. This makes the third transplantation a surgical challenge as a third dissection of the iliac fossa could pose surgical risks such as higher volume of blood loss, increased risk of iliac vessel injury, increased warm ischemic time, and subsequently, a prolonged duration of surgery [20]. A third KT is best performed intraperitoneally [16], although some authors opined that a retroperitoneal approach to the iliac fossa should be considered as a first choice [20]. Others suggest the subhepatic retroperitoneal space [6,21]. Ultimately, a rigorous patient work-up that includes a CT angiography of the vessels and the skills of the surgeon are the primary determinants of the success of the procedure.

When compared with patients who are seronegative, patients with hepatitis B infection have worse patient and graft outcomes [22]. In the post-transplant period, the severity of hepatitis B infection increases, in part due to the immunosuppressants used by post-transplant patients to prevent allograft rejection. Antiretroviral therapy aimed at effectively suppressing viral replication significantly reduces this risk, coupled with effective viral load monitoring.

In Nigeria, renal replacement therapy is not covered by the national health insurance scheme (NHIS), and the financial burden of getting a kidney transplant for patients living with ESKD is borne by the patient and/or their family and friends [23]. Only a select few private health insurance companies and health maintenance organizations (HMOs) support patients with renal replacement therapy. These few HMOs rarely fund repeat KTs. The chances of an ESKD patient getting funds to undergo a KT more than once are slim; our patient was an exception.

Since we only followed up with our patient for a short time before writing up this case, we cannot report on the medium- and long-term effects of the procedure. This is a limitation of this study.

## Conclusions

Third kidney transplantation can be performed successfully despite the challenges of HLA sensitization, antibiotic resistance, and surgical placement of the graft. Key considerations for success include effective desensitization, patient optimization, and careful surgical planning. Kidney transplant centres in west and central Africa struggle with the high cost of transplant (including desensitization) and surgical skill, which makes a third transplant difficult.
